# Knockout of Ku86 accelerates cellular senescence induced by high NaCl

**DOI:** 10.18632/aging.100022

**Published:** 2009-02-12

**Authors:** Natalia I. Dmitrieva, Hua Tang Chen, André Nussenzweig, Maurice B. Burg

**Affiliations:** ^1^Laboratory of Kidney and Electrolyte Metabolism, National Heart, Lung, and Blood Institute, National Institutes of Health, Bethesda, MD 20892, USA; ^2^Experimental Immunology Branch, National Cancer Institute, National Institutes of Health, Bethesda, MD 20892, USA

**Keywords:** salt, DNA damage, osmotic stress, senescence, aging, dehydration, Ku86 mice

## Abstract

NaCl induces DNA breaks, thus leading to cellular senescence. Here we showed that Ku86 deficiency accelerated the high NaCl-induced cellular senescence. We find that 1) high NaCl induces rapid cellular senescence in Ku86 deficient (xrs5) cells, 2) Ku86 deficiency shortens lifespan of *C. elegans* in high NaCl, and 3) cellular senescence is greatly accelerated in renal inner medullas of Ku86^-/-^ mice. Further, although water balance is known to be compromised in old mice, this occurs at much earlier age in Ku86^-/-^ mice. When subjected to mild water restriction, 3 month old Ku86^-/-^, but not Ku86^+/+^, mice rapidly become dehydrated as evidenced by decrease in body weight, increased production of antidiuretic hormone, increased urine osmolality and decreased urine volume. The deficiency in water balance does not occur in Ku86^+/+^mice until they are much older (14 months). We conclude that Ku86 deficiency accelerates high NaCl-induced cellular senescence, particularly in the renal medulla where NaCl normally is high.

## Introduction

High NaCl induces DNA breaks and oxidative damage to DNA and proteins  [1-3], and also promotes cellular senescence both in cell culture and *in vivo* [4]. Despite these changes, the cells proliferate at a close to normal rate in culture and maintain their function in renal inner medullas *in vivo* where NaCl is normally high. Evidently, there must be mechanisms that promote survival and function of cells despite the seemingly adverse high NaCl-induced changes. Ku86 is important in this respect. It binds to the ends of DNA that is broken following ionizing radiation (IR) and during V(D)J recombination, and it facilitates DNA repair by aligning DNA ends for non-homologous end joining (NHEJ) [5]. We previously found that Ku86 deficiency compromises adaptation of cells to high NaCl [6]. This was most dramatic in the radiosensitive xrs5 mutant cell line, derived from CHO-K1 cells by treating them with ethyl methanesulphonate, resulting in Ku86 deficiency [7,8].  These cells never recover from the initial cell cycle arrest induced by high NaCl. They lose their epithelial appearance, become giant and multinucleated, and disintegrate within 10 days after NaCl is raised to a level that normal cells adapt to readily. Spontaneously immortalized mouse embryonic fibroblasts (mefs) from Ku86^-/-^ mice do proliferate despite high NaCl, but their growth rate is greatly reduced compared to Ku86^+/+^ mefs.  The number of broken chromosomes is greater in Ku86^-/-^ mefs exposed to high NaCl than in Ku86^+/+^ mefs [6]. Since these high NaCl-induced changes that occur in Ku86^-/-^ cells resemble those known to be associated with cellular senescence [9-11], we have in the present studies tested the hypothesis that Ku86 deficiency might accelerate the cellular senescence induced by high NaCl.

## Results

### xrs5 (Ku86 deficient) cells undergo rapid senescence when NaCl is elevated

**Figure 1.**
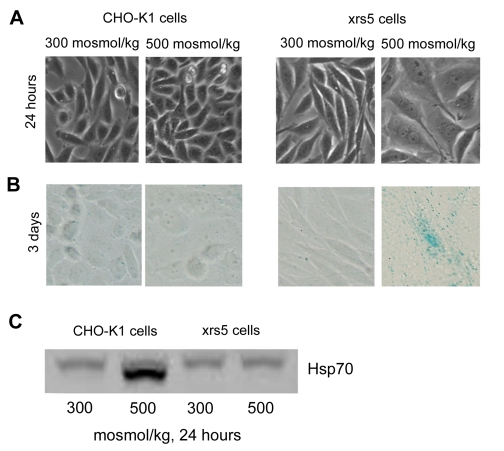
High NaCl induces rapid senescence of Ku86 deficient (xrs5) cells. Medium bathing CHO-K1 (wild type) and xrs5 (ku86 mutant) cells grown at 300 mosmol/kg was acutely changed to the same medium or to 500 mosmol/kg (NaCl added). (**A**) Photographs after 24 hours. High NaCl rapidly induces cellular hypertrophy in xrs5 cells.  (**B**) Staining for senescence-associated β-galactosidise (SA-β-gal). Positive staining for SA-β-gal is detected 3 days after NaCl elevation. (**C**) Western blot for Hsp70 expression. Hsp70 is not upregulated  in xrs5 cells exposed to high NaCl, consistent with senescence.

In our previous studies we found that xrs5 cells undergo dramatic morphological changes upon exposure to high NaCl. They change from epithelial to fibroblast morphology, enlarge, flatten and become multinucleated [6]. To test whether the cell have become senescent, we stained them for expression of senescence associated β-galactosidase (SA-β-gal). We confirm that within 3 days of exposure to high NaCl the morphology of xrs5 cells changes dramatically (Figure 1A) and now find that, in addition, they become positive for SA-β-gal (Figure 1B), indicative of senescence. In contrast, the appearance of the control CHO-K1 (wild type) cells does not change (Figure 1A) and they do not express SA-β-gal (Figure 1B). Also, we find diminished expression of HSP70 in response to high NaCl, which is an additional indication of senescence since, although high NaCl increases expression of HSP70 [12], senescence reduces it [13,14].

High NaCl increases expression of HSP70 in CHO-K1 cells, but not in xrs5 cells (Figure 1C), providing an additional indication that high NaCl induces senescence in Ku86 deficient cells.

### Exposure to high NaCl, starting in the larval stage, causes a greater reduction of the life span of *C. elegans* that lack Ku86 activity than of wild type

**Figure 2.**
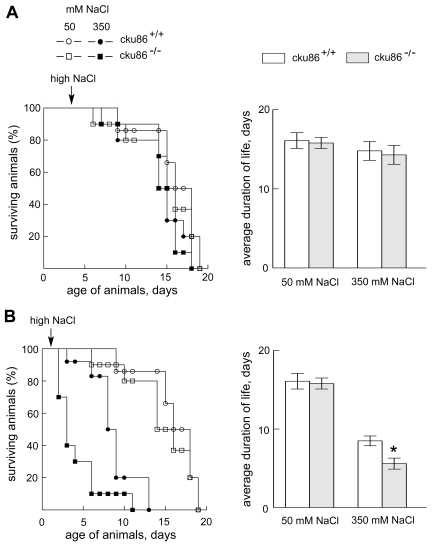
Absence of Ku86 reduces longevity of *C. elegans* in high NaCl, provided the exposure to high NaCl begins in the larval stage. *C. elegans* were placed on plates containing 50mM or 350 mM of NaCl beginning at the (**A**) adult stage (day 4 after hatching) or (**B**) L2/L3 larva stage (day 2 after hatching). Every two days worms were transferred to new plates to separate them from their progeny. Left panels: % of animals surviving. Right panels: average duration of life (mean ±SEM, * P < 0.05 relative to control (cku86^+/+^).

Previously, we showed that exposure of *C. elegans* to high NaCl accelerates accumulation of senescent cells and decreases their life span. In the present studies we tested whether lack of Ku86 activity further diminishes longevity in the presence of high NaCl. We compared the effect of high NaCl on wild type *C. elegans* to that on cku80^-/-^ *C. elegans*, which lack activity of the Ku86 homologue. If NaCl is first elevated when the animals are adults (4 days old), life span is little affected and does not differ between the mutants and the wild type (Figure 2A). In contrast, if NaCl is first elevated while they are larvae (2 days old), life span decreases markedly and the life span of the mutants is significantly less than the wild type (Figure 2B). Since somatic cells of C. elegans do not proliferate once they reach adult stage [15], the difference may lie in greater susceptibility to the effect of high NaCl of proliferating cells in the larvae.

### Knock out of Ku86 accelerates cellular senescence in the mouse renal inner medulla *in vivo*

**Figure 3.**
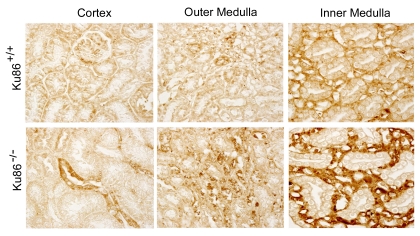
Immunocytochemical analysis of p16 ^INK4^ in kidneys of 3 month old Ku86^+/+^    and Ku86^-/-^ mice. Many senescent cells (brown stain) are present in kidneys of Ku86^-/-^ mice. p16^INK4^ is higher in the renal medulla, where salt concentration normally is always high, than in the cortex, where the salt concentration is similar to that in peripheral blood. p16^INK4^ level and staining pattern in kidneys of 3 month old Ku86^-/-^ mice are similar to those observed previously in 12 month old wild type mice [4].

We next tested whether absence of Ku86 makes mouse renal cells more prone to high NaCl-induced cellular senescence *in vivo*, using expression of the cell cycle regulator p16^INK4^ as an indicator [16,17]. NaCl normally is always high in the renal inner medullary interstitium associated with its role in the urinary concentrating mechanism, but it is not high in the renal cortex. Using this assay, we previously found only low levels of cellular senescence in both the renal cortex and medulla at 3 months of age. At 12 months expression becomes high in the medulla, but not in the cortex [4]. In the present studies we confirm that in Ku86^+/+^ mice p16^INK4^ is not elevated in either renal inner medulla or cortex at 3 months (Figure 3). In contrast, the renal medullas, but not cortex, of Ku86^-/-^ mice already contain numerous senescent cells at this age (Figure 3). Thus, absence of Ku86 greatly accelerates accumulation of senescent cells in the renal inner medulla. As previously noted [4], it is not renal medullary epithelial cells that become prematurely senescent, but adjacent cells that surround the tubules.

### The deficit in water conservation that occurs normally in old mice, occurs at an earlier age in Ku86^-/-^ mice

**Figure 4.**
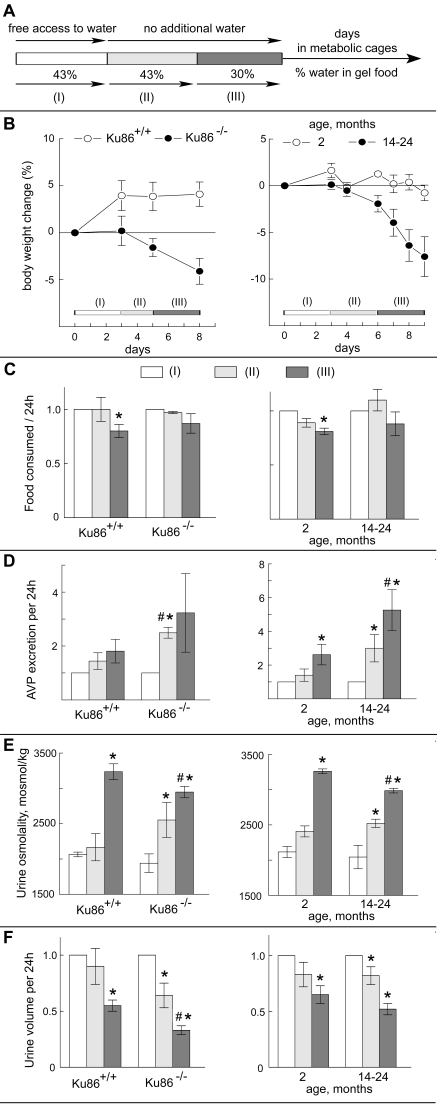
Effects of Ku86 deficiency and aging on water conservation. The experiment analyzes the response to mild water restriction of 3 months old Ku86^+/+^    versus Ku86^-/-^ mice (left panels) and of 2 months old versus 14-22 months old mice (right panels). (**A**) Experimental design.  Mice were subjected to 3 consecutive periods of different water availability. During period I mice had free access to water and the gel food containing 43% of water. Then, the supplemental drinking water was removed and mice got water only from the gel food (period II). During period III, the amount of water in the gel food was decreased to 30%. The periods lasted 3 days, except period (II) for Ku86^+/+^ and Ku86^-/-^ mice which lasted 2 days. Body weight (**B**), food consumption (**C**), Arginine Vasopressin (AVP) excretion (**D**), urine osmolality (**E**) and urine volume (**F**) were measured every 24h. Average values during each period were calculated and normalized to period (I) for the same mouse. Data are represented as mean ±SEM (n=3-5, * P < 0.05 relative to period I, # P<0.05 relative to the same period in the parallel group).

Antidiuresis, which depends on intact function of the renal medulla, is important for water conservation. Aged subjects are prone to dehydration  [18,19]. The following experiments were aimed at 1) finding if old mice have a deficit in water conservation, 2) if so, whether it occurs prematurely in Ku86^-/-^mice, and 3) whether any deficit involves defective urinary concentrating ability. We analyzed the response of Ku86^+/+^ versus Ku86^-/-^mice and of mice of various ages to mild water restriction. The mice were maintained in individual metabolic cages. Their food was in the form of a gel, maintaining a constant amount of dry food, but variable water content (Figure 4A). The experiment was divided into three periods: (I) gel food containing 43% water, plus free access to drinking water; (II) and (III) no additional drinking water; and (III) water content of the gel food reduced to 30% (Figure 4A).  Body weight, food consumption, urine volume, urine osmolality, and urinary vasopressin excretion rate were measured. Data are expressed relative to period (I). Experiments were of two sorts: Ku86^+/+^ versus Ku86^-/-^mice at 3 month of age (Figure 4, left panels) and 2 month old versus 14-24 month old wild type mice (Figure 4, right panels). Changes in body weight (Figure 4B) are an index of fluid balance since consumption of dry food (Figure 4C) either did not change significantly (Ku86^-/-^ and 4-24 month old) or varied slightly, uncorrelated with weight changes (Ku86^+/+^ and 2 month old). Three month old Ku86^+/+ ^(Figure 4B, left panel)and 2 month old wild type mice (Figure 4B, right panel) do not lose weight during the mild water restriction in periods (II) and (III). Evidently, they can regulate their water balance to avoid net loss when water is restricted. In contrast, the Ku86^-/-^ (Figure 4B, left panel) and 14-24 month old (Figure 4B, right panel) mice lose weight rapidly, indicating that ability to maintain water balance decreases with age and that lack of Ku86 accelerates the process. Greater excretion of antidiuretic hormone (ADH, Figure 4D) provides additional evidence that Ku86^-/-^ and 14-24 month old mice become more dehydrated than Ku86^+/+^ and 2 month old mice following water restriction.

Since intake of water (limited to the gel food) is fixed during  periods  (II)  and  (III), the rapid weight loss of Ku86^-/-^ mice and 14-24 month old mice must be due to water loss. Given the evidence of senescence in the renal medullas of these animals, our first thought was that the water loss might be due to inability to concentrate their urine sufficiently. However, the Ku86^-/-^mice reduce water excretion in their urine even more than do the Ku86^+/+ ^mice (Figure 4F, left panel) and the 14-24 month old mice reduce their urine volume at least as much as do the 2 month old mice Ku86^+/+ ^(Figure 4F, right panel), so excess loss of water in the urine is not the explanation. Also, both the Ku86^-/- ^and 14-24 month old mice make urine that is highly concentrated (albeit slightly less than the Ku86^+/+^ 2 month old mice) in response to water restriction (Figure 4E). Thus, poorly regulated urinary loss does not account for the deficient water balance in these animals. The alternative is poorly regulated extrarenal loss of water. However, our present experiments do not identify the route of such loss.

We conclude that old mice do not conserve water as well as young mice, apparently due to poorly regulated extrarenal loss, and that the deficiency occurs at an earlier age in mice that lack Ku86.

## Discussion

### High NaCl promotes cellular senescence

We previously found that exposure to high NaCl promotes cellular senescence [4]. The evidence included that: 1) Chronic exposure to high NaCl induces senescence in HeLa cells and accelerates senescence of primary mefs. 2) Elevated NaCl reduces the life span of *C. elegans*, while increasing the number of senescent cells. 3) Cells become senescent much faster *in vivo* in mouse renal inner medullas, where they are normally exposed to elevated interstitial NaCl, than in the renal cortex where they are not. High NaCl causes DNA damage and oxidative stress [2,20], which are known precursors of cellular senescence [9].

### Ku86 deficiency accelerates high NaCl-induced cellular senescence in cultures

Cells in culture adapt to high NaCl despite the presence of a continuously increased number of  DNA breaks. This evidently requires some mechanism for maintaining chromatin integrity. We previously found that Ku heterodimers are important in this respect, presumably because they bind to broken ends of DNA and align them [6]. Thus, high NaCl fragments chromosomes more in Ku86^-/-^ than in Ku86^+/+ ^mefs. In addition, high NaCl reduces the rate of proliferation of Ku86^-/-^ more than Ku86^+/+ ^mefs, and a senescent morphology appears, including cellular enlargement and flattening [6]. The changes are even more striking in xrs5 cells, which were derived by ethyl methane-sulphonate mutation of CHO-K1 cells, resulting in loss of Ku86 [7,8]. Upon exposure to high NaCl, these cells enlarge, flatten and become multinucleated within 2 days, and their cell cycle becomes permanently arrested [6]. Since these are morphological changes characteristic of cellular senescence, we tested for that specifically in the present studies by using SA-β-gal, which is a marker of cellular senescence. We find that by day 3 of exposure to high NaCl the cells do become positive for SA-β-gal (Figure 1), consistent with a role for Ku86 in delaying high NaCl-induced senescence.

### Ku86 deficiency accelerates high NaCl-induced cellular senescence *in vivo*

Since senescence pathways are modified in immortalized cells [21,22] we conducted *in vivo* experiments to test whether Ku86 protects normal cells from high NaCl-induced cellular senescence. In previous studies we found that high NaCl accelerates accumulation of senescent cells and decreases longevity of *C. elegans* [4]. Absence of cku86 further decreases longevity of *C. elegans* exposed to high NaCl (Figure 2), consistent with a role of Ku86 in delaying NaCl-induced senescence. It is of interest that the age at which *C. elegans* are first exposed to high NaCl critically determines its effect. NaCl reduces longevity of *C. elegans* only if they are first exposed to it as larvae (2 days after hatching), not if they are first exposed to it as adults (4 days after hatching) (Figure 2). A possible explanation is that somatic cells of adult *C. elegans*, being postmitotic and unable to divide [15], are not affected, while the dividing cells in the larvae are affected. The only proliferating cells in adult *C. elegans* are contained in the gonads and embryos in their reproductive tract, and those cells apparently are affected by exposure to high NaCl. High NaCl decreases the number of progeny from wild type *C. elegans* and the decrease is even greater in cku86 *C. elegans* [6].

We also tested whether knockout of Ku86 might accelerate high NaCl-induced cellular senescence in the kidney *in vivo*. NaCl is normally elevated in renal inner medullary interstitial fluid, which powers the urinary concentrating and diluting mechanisms. It is not elevated in the renal cortex. We previously found that in 12 month old mice there are many more senescent cells in the inner medulla than in the cortex [4]. In the present studies we tested younger mice. We found that at 3 months of age there are already many more senescent cells in the inner medullas of Ku86^-/-^ mice than in Ku86^+/+^mice. We conclude that Ku86 delays the appearance of high NaCl-induced senescence in mouse renal inner medullas *in vivo*.

### Does high NaCl-induced cellular senescence contribute to early aging of Ku86^-/-^mice?

Ku86^-/-^mice age prematurely [23,24]. They also have defective NHEJ DNA repair, severe combined immunodeficiency (scid) [25,26], and chronic inflammation. These other defects have been considered as possible causes of the accelerated aging. However, immunodeficiency, alone, apparently is not the cause since mice deleted for Rag-1, also suffer from scid and chronic inflammation, but do not age prematurely [27]. Similarly, defective NHEJ, alone, apparently is not the cause because defects in another NHEJ protein, DNA-PKcs, do not cause prominent premature aging [28,29]. Having noted that Ku86^-/-^ mice are susceptible to dehydration from even a very limited restriction of water (Figure 4), we were led to wonder whether they might be chronically dehydrated enough to raise their blood NaCl sufficiently to contribute to premature cellular senescence and aging. Old age is associated with dehydration [18,19,30]. The mechanisms implicated include decreased thirst, which leads to insufficient water intake and impaired renal response to ADH, which leads to excessive loss of water in the urine. (reviewed in [18,19]) We find that water conservation is impaired in old mice and that Ku86 deficiency accelerates the impairment (Figure 4), like it accelerates other aspects of aging. Interestingly, water balance is impaired in Ku86^-/-^ mice much earlier than other aspects of aging, including kyphosis and premature closure of growth plates. Thus, 3 month old Ku86^-/-^ mice already have impaired water conservation (Figure 4), whereas kyphosis does not occur until 6 months of age and premature growth plate closure until 5 months [23]. Thus, impaired water conservation could be contributing to other aspects of premature aging in Ku86^-/-^ mice.

## Methods

Cell culture. Xrs5 (X-ray sensitive Chinese Hamster Ovary, no.CRL-2348, American Type Culture Collection, Manassas, VA) is a mutant cell line which was derived from CHO-K1 cells (no.CCL-61, American Type Culture Collection) by treating the cells with ethyl methanesulphonate. These cells belong to X-ray complementation group 5 and are mutant in the p86 subunit of the Ku autoantigen [7,8]. We grew the cells in DMEM plus 10% fetal bovine serum (HyClone, Logan, UT). Osmolality of control ("isotonic") medium was 300 mosmol/kg. High NaCl medium was prepared by adding NaCl to the total osmolality indicated.

Staining of cells for SA-β-gal activity. The Senescence β-Galactosidase Staining Kit (Cell Signaling, Beverly, MA) was used, as previously described [4]. Senescent cells are indicated by blue color.

*C. elegans* strains and culture *.* Bristol N2 (Wild type) and *cku80 (ok861)* C. elegans were provided by *Caenorhabditis* Genetic Center (CGC, Minneapolis, MN). The *cku80(ok861) *strain contains a homozygous 1,646-bp deletion, including a large section of coding sequence, in the *cku80* locus. The deletion was confirmed by PCR in our previous publication [6]. The worms were grown on Nematode Growth Medium agar plates spread with *E. coli* strain OP50 (obtained from CGC). Cultures were maintained at room temperature (about 20^◦^C). Control Nematode Growth Medium contains 51mM NaCl, 1mM MgSO_4_, 1mM CaCl_2_, 25mM KPO_4_, 5μg/ml cholesterol, 2.5g/l peptone, and 17g/l agar [31]. We increased NaCl by adding 300 mM, as indicated. To measure longevity we transferred L2-L3 larvae or adult *C. elegans* to control or high NaCl agar plates. Every other day the original worms were transferred to new plates to separate them from their progeny. The number surviving was counted every day. Worms were considered dead if they did not respond to repeated prodding with a platinum wire.

Immunohistological detection of p16Ink4 in kidney sections. Mouse kidneys were fixed overnight in 4% paraformaldehyde at 4°C, and then embedded in paraffin. Sections were cut and mounted on silanized slides by American Histolabs (Gaithersburg, MD). Sections were stained with anti-p16 (sc-1207: Santa Cruz, Santa Cruz,   CA) as previously described [4]. A Nikon E800 Widefield Microscope was used for photography.

Measurement of water balance. The Ku86-/- mice used in this study were previously described [25]. Wild type mice were purchased at age of 2-3 months from Taconic (129S6, Model no.129SVE, Taconic Farms, Inc, Hudson, NY) and housed in the NHLBI animal facility. All mouse studies were done under approved National Heart, Lung, and Blood Institute and National Cancer Institute animal study protocols and mice were housed in an Association for Assessment and Accreditation of Laboratory Animal Care-accredited facilities. Mice were maintained in mouse metabolic cages (Hatteras Instruments, Cary, NC) during the study under controlled temperature and light conditions (12-h light and dark cycles).

The experiment design is shown on Figure 4A. Initially, all mice received gelled food containing 43% of water. The gelled food contained 3 ml of deionized water, 4 g of balanced purified rodent diet (AIN-76A, Research Diets, New Brunswick, NJ), and 70 mg of agar per 7 g of the food. Food in the metabolic cages wasprovided in excess so the mice could eat what they wanted. Drinking water was provided ad libitum during this period. After 2 days of adaptation, mice were subjected to 3 consecutive periods of differing water availability (Figure 4A). During period I mice had free access to water and the gel food containing 43% water. Then, supplemental drinking water was removed so the only water was that contained in the gel food (period II). During period III, the amount of water in gel food was decreased to 30% (1.7ml of water, 4g of the rodent diet powder and 57 mg of agar). Body weight, urine volume, food consumption, urine osmolality and urine Arginine Vasopressin (AVP) concentration were measured every 24h. Urine was collected under mineral oil in pre-weighed collection vials. Urine volume was measured gravimetrically, by assuming a density of one. Gel food was supplied in preweighed plastic cups to facilitate measurement of consumed food. Urine osmolality was measured using Fiske Model 210 Freezing-Point Micro-Osmometer (Fiske Associates, Norwood, MA). AVP concentration in urine was measured using Vasopressin Enzyme Immunoassay Kit (no. 900-017, Assay Designs, Ann Arbor, MI).

Statistics. Average values during each period were normalized to period (I). Data were evaluated by t-test, paired t-test comparison to period (I), unpaired t-test for comparison between groups. A p-value less than 0.05 was considered significant.
